# Research on the quality supervision strategy of drugs sold on the internet under the MAH system

**DOI:** 10.3389/fpubh.2025.1457340

**Published:** 2025-04-09

**Authors:** Zongliang Wen, Jialin Chen, Li Zhou, Long Bai, Shenqin Wu, Yun Zhao, Jinhua Fang

**Affiliations:** ^1^School of Management, Xuzhou Medical University, Xuzhou, China; ^2^Institution of Chinese Health Modernization, Xuzhou Medical University, Xuzhou, China; ^3^Affiliated Hospital of Xuzhou Medical University, Xuzhou, China; ^4^School of Public Health, Nanjing Medical University, Nanjing, China; ^5^School of Public Health, Xuzhou Medical University, Xuzhou, China

**Keywords:** drug quality supervision, MAH system, online sales of drugs, evolutionary game, simulation analysis

## Abstract

**Background:**

In recent years, purchasing medications online has become increasingly popular. However, occasional quality issues have arisen with drugs bought online. As a result, we need effective quality monitoring of medicines sold online. To address this issues, several countries have begun to implement the Marketing Authorization Holder (MAH) system to enhance drug quality control.

**Methods:**

Consequently, this paper develops a four-party evolutionary game model that includes the government, holder, agent seller and third-party platform in pharmaceutical online sales, and use Matlab 2022b to carry out numerical simulation, and to compare and analyze the influence of different factors on the strategy selection of the main body of the game by changing parameters. This paper is based on the multi-party game perspective, constructed a hierarchical model, thereby better exploring the mechanism of drug quality regulation in the context of drug online sales under the MAH system.

**Results:**

Findings suggest that reducing operational costs motivates agent sellers to adopt stable, honest strategies. Increased government penalties expedite the adoption of positive strategies by sellers, holders, and third-party platforms. Enhanced penalties from holders and platforms for defaults effectively regulate seller behavior. Additionally, reputation rewards or losses incentivize sellers to adopt honest practices, with consumer and sellers' reputations influencing the likelihood of comprehensive third-party platform reviews.

**Conclusions:**

In summary, policy makers should improve the regulatory mechanism, establish a fair system of rewards and penalties, develop a compensation mechanism for information sharing, deregulate prices and enhancing feedback channels.

## 1 Introduction

The rapid evolution of the Internet and the pharmaceutical industry, particularly amid epidemic outbreaks, accelerates the growth of online drug businesses. The increasing convenience of purchasing drugs online, coupled with the high incidence of covert illegal activities, the swift pace of industry development, and serious safety concerns, has led to a continuous emergence of global drug quality and safety incidents in recent years (1). In 2018, the U.S. FDA (Food and Drug Administration) closed down more than 1,600 illicit online pharmacies in the U.S. for distributing counterfeit medicines, highlighting regulatory loopholes and the global reach of unlawful practices facilitated by the Internet (2). China continues to grapple with the persistent issue of counterfeit and unlicensed medications being widely available on e-commerce platforms, facing challenges in efficiently regulating the online pharmaceutical market (3). Similarly, in India, relaxed regulations have spurred the proliferation of unlicensed online pharmacies selling substandard medications, posing public health risks (4). These instances illustrate issues in the current online sale of medicines, including the use of the Internet for the sale of counterfeit medicines, illicit sale of prescription drugs, lack of standardized information display, variability in product types, and misleading advertisements. This further indicates that the current regulation of online drug sales lacks a robust legal foundation, with superficial and rudimentary supervision and inspection and a traditional, singular approach to supervision (5, 6). Countries are formulating strategies or initiatives to enhance the efficiency and effectiveness of drug quality regulation to safeguard public health, safety, and pharmaceutical quality in the supply chain (7).

In 2019, China introduced a new Marketing Authorization Holder (MAH) system for drugs to foster the growth of the pharmaceutical sector and guarantee the quality and safety of medications (8). With the progress of Internet technology and the rapid expansion of the digital economy, online medicine sales have become a crucial avenue for patients to procure medications. Within the online sphere, drug quality oversight involves governmental bodies, holders, manufacturers, sellers, third-party platforms, and other entities, influencing the quality supervision approach in online drug retailing (9). However, the existing drug quality supervision framework remains imperfect, necessitating enhanced enforcement of responsibilities among all stakeholders and bolstered oversight of online medicine sales.

This article employs multi-body evolutionary game theory to examine various real-life scenarios involving the government, holders, entrusted producers, agent sellers, and third-party platforms to determine the stable equilibrium point of strategy selection among multiple entities in the game and analyze the roles' mechanism in diverse scenarios (10). It aims to address the following three questions: **First, does the adoption of different strategies by each subject have an impact on other subjects? If yes, what is the precise nature of this impact? Second, how should the government, holders, sellers, and third-party platforms collaborate to ensure the quality standards of drugs sold online?**

The structure of the article is as follows: The second part compiles and reviews relevant literature, and the third part establishes an evolutionary game model involving the government, holders, producers, sellers, and third-party platforms. The fourth part analyzes the stability of the game strategies of the four entities, while the fifth part examines the stability of the equilibrium point of the multi-subjects decision-making behaviors using Lyapunov's first method. The sixth part involves the simulation analysis in MATLAB 2022b, the seventh part discusses and proposes relevant suggestions, and the paper concludes.

## 2 Relevant literature

### 2.1 Marketing Authorization Holder

The MAH (Marketing Authorization Holder) system originated in Europe and the United States, and its core lies in separating the marketing authorization of drugs from the manufacturing authorization (11). In the late 20th century, with the rapid development of the biopharmaceutical industry, Europe and the United States passed legislation to establish the MAH system, which allows R&D institutions, natural persons, and other non-manufacturing enterprises to hold the marketing authorization for drugs and entrust qualified enterprises to manufacture them (12). The introduction of the MAH system aims to improve the efficiency and quality of drug management and ensure the quality and safety of drugs by clarifying the responsible parties. Developed countries such as the United States, the European Union, and Japan have relatively well-established laws and regulations governing drug marketing applications, approvals, and licensing (13, 14).

The European Union has granted MAHs the right to sell across borders through the Centralized Procedure, which requires holders to set up pharmacovigilance systems and assume responsibility for monitoring adverse reactions (15). The U.S. FDA implements the MAH system, emphasizing the holder's responsibility for the quality, safety, and efficacy of drugs, including the commissioning of the production of quality control agreements and supply chain traceability system (16, 17). Japan and Singapore have attracted international R&D resources through the MAH system to promote the upgrading of the local biopharmaceutical industry. For example, Japan allows foreign companies to act as MAHs, but they are required to designate a local agent to be responsible for compliance matters (18).

However, in China, the development of the MAH system has been relatively slow. The system was initiated as a pilot program in certain regions in 2016 and was fully established only in 2019 (19). With the implementation of the MAH system, the scope of commissioned production and sales has expanded, allowing various holders the right to commission drugs. However, the quality of these commissioned drugs varies. Most existing studies are centered on commissioned production, with fewer addressing the problems of commissioned sales and the regulatory mechanisms involved.

### 2.2 Online sales of drugs

The frequent incidents of drug safety and quality issues worldwide in recent years highlight numerous deficiencies in the regulatory mechanisms governing drug quality, particularly during the developmental phase of online pharmaceutical businesses. In developed countries, there has been an earlier emphasis on scientifically regulating medications, resulting in a relatively robust and healthy development of online pharmaceutical sales. Research in Western regions has indicated that drug regulatory bodies should resist compromising standards in pursuit of affordability and explore ways to maintain the sustainability of drug expenditures (20). Effective Drug regulatory policies can improve medicine quality, drive pharmaceutical innovation, and enhance the global competitiveness of firms (21).

In contrast, research by Chinese scholars on the online pharmaceutical industry primarily highlights the absence of a legal foundation for governmental oversight of online transactions and a reliance on traditional and singular regulatory strategies (22). There is an urgent need to adopt emerging technologies to enhance regulatory strategies and establish a network information-sharing mechanism to facilitate collaborative regulation across multiple departments (23).

The drug regulatory landscape differs significantly between China and other countries due to historical, cultural, and economic factors. While China's domestic regulations are evolving toward a more transparent and efficient framework, foreign regulatory bodies such as the FDA and EMA have established processes underpinned by comprehensive guidelines and robust enforcement mechanisms (24). Strengthening regulatory frameworks and ensuring the safety and efficacy of drug development remain shared global objectives that necessitate ongoing dialogue and collaboration among nations (25). Given the multiple stakeholders involved in the online drug business, further research is needed to explore effective methods for controlling the quality of online drugs and implementing collaborative regulatory strategies.

In summary, existing literature on the Marketing Authorization Holder (MAH) system and the online drug business commonly utilizes comparative analysis and survey research to assess the current status and challenges and suggest potential improvement strategies. However, there is a scarcity of research on the stability of drug quality supervision strategies within the context of game theory, with the majority of studies focusing on analyzing the relational dynamics of two or three entities within specific aspects of drug quality supervision. The implementation of the MAH system and the advancement of online drug business have expanded the scope of stakeholders engaged in drug quality supervision. The holder assumes accountability for the entire drug quality process, and the third-party platform is also tasked with monitoring drug quality.

**Figure 1:**
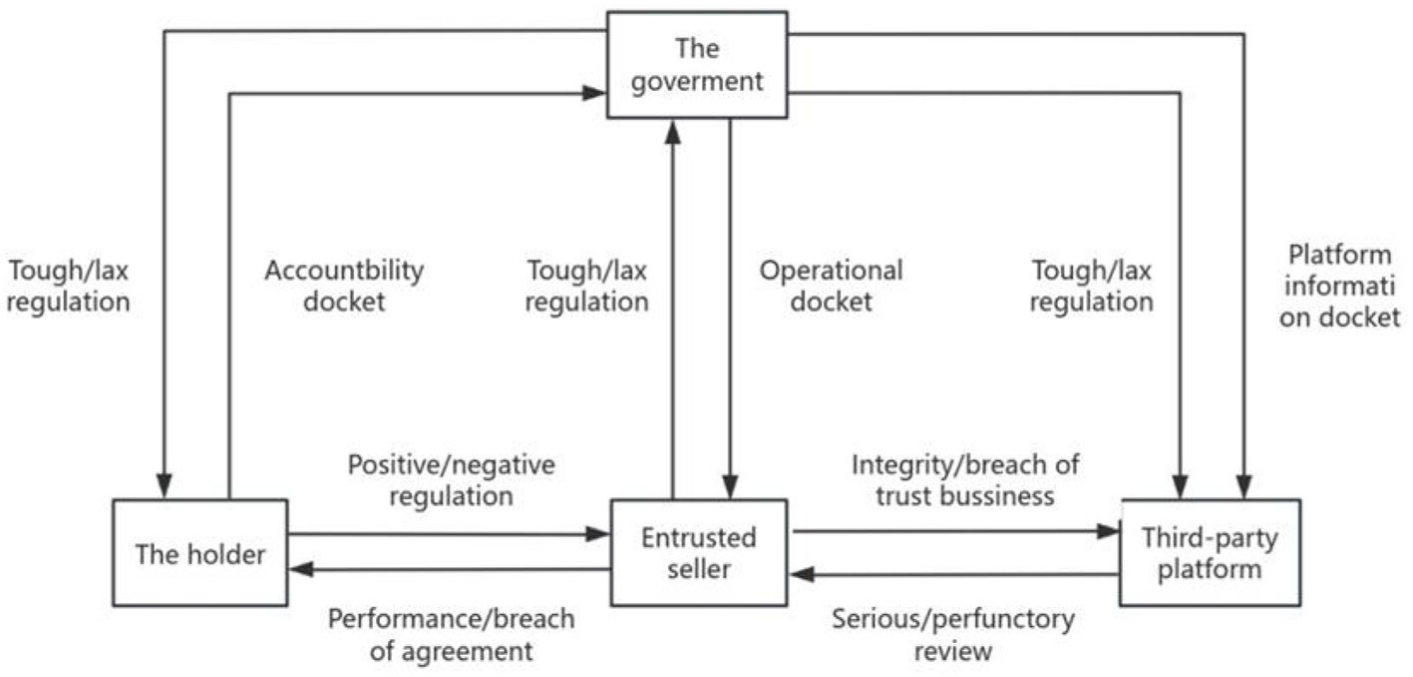
Government–holder–agent seller–third party platform game logic relationship diagram.

This study selects the government, the holder, the entrusted seller, and the third-party platform as the game subject and constructs the four-party game model in the process of pharmaceutical network sales; its game model logical relationship is shown in Figure 1.

## 3 Model

This article explores the design of quality supervision strategies for online drug sales under multi-party collaborative supervision. It constructs a game model of multi-party participation in drug quality supervision based on evolutionary game theory and proposes corresponding suggestions for the quality supervision of online drug sales under the MAH system.

### 3.1 Model assumptions

#### 3.1.1 Assumption 1

In the evolutionary game, the government, the holder, the trustee-seller, and the third-party platform are all limited rational subjects; out of the consideration of maximizing their interests, their strategic choices will tend to be optimal with the evolution of time. The probability that the government department chooses to regulate severely is *e*, and the possibility of lax regulation is 1−*e*. The possibility of the holder adopting positive regulation is *f*, and the possibility of negative regulation is 1−*f*. The possibility of the agent seller choosing to operate in good faith is *g*, and the possibility of choosing to operate in bad faith is 1−*g*. The possibility of third-party platforms adopting careful scrutiny is *h*, and the possibility of a perfunctory review is 1−*h*.*e, f, g, h*∈[0, 1].

#### 3.1.2 Assumption 2

The cost to the government departments for stringent regulatory measures is *C*_*e*_. When the agent seller chooses to operate in good faith, the pharmaceutical industry develops in an orderly manner, public medication safety is guaranteed, and the government will gain social benefits *R*_*e*_. When a seller's breach of trust in business disrupts the market order and damages public health and property, the government needs to spend energy and financial resources to repair the adverse effects caused, and the government will face social losses *D*_*e*_. When the government adopts stringent regulatory measures, it will penalize negative regulatory holders, sellers operating in breach of trust, and third-party platforms conducting perfunctory reviews on a case-by-case basis, with fines amounting to *F*_*f*_, *F*_*g*_, and *F*_*h*_, respectively.

#### 3.1.3 Assumption 3

The cost to the holder in taking active regulatory measures is*C*_*f*_. According to the consignment sales agreement, the holder delivers the pharmaceutical products of acceptable quality to the consigned seller at a specified quantity and price and receives a fixed income *R*_*f*_. If the holder finds out that the agent seller has failed to operate in accordance with the agreement, the holder may withdraw its right of agency and demand payment *Ig*_1_ according to the agreement when taking active supervision measures.

#### 3.1.4 Assumption 4

The cost to the trustee-seller of operating in good faith is *Cg*_1_, and the cost to the trustee-seller of operating in bad faith is *Cg*_2_, *Cg*_1_>*Cg*_2_. Assume that the benefit to the commissioned seller is *R*_*g*_, where *R*_*g*_ has been subtracted from the purchase amount *R*_*x*_ and other costs. When the agent seller operates in good faith, the good image will bring the enterprise reputation value premium *S*_*g*_ (e.g., expanding the scope of the agency, patients' trust, etc.); when the agent seller fails to operate in good faith, the enterprise will be faced with the negative evaluation of the patients and demand for corresponding financial compensation, resulting in the loss of reputation *D*_*g*_.

#### 3.1.5 Assumption 5

Assume that the third-party platform can earn revenue *R*_*h*_ from its operation and the cost of adopting careful scrutiny is *C*_*h*_. When a patient suffers a health loss due to a breach of trust in the operation of the enterprise, the third-party platform will also face compensation *D*_*h*_ to the patient. When the third-party platform takes a serious review, it can stop providing online trading platform services to the defaulting enterprise and require it to pay the compensation amount *Ig*_2_ for the loss of the platform. At the same time, it also protects the operation of other honest enterprises and the interests of patients, which can increase its reputation value *S*_*h*_.

The parameters and descriptions of this article are shown in Table 1.

**Table 1:** Related parameter description.

**Parameter**	**Description**	**Parameter**	**Description**
*e*	Probability of strict regulation by the government	*S*_*g*_	Reputation premium for the agent seller
*f*	Probability of the holders to adopt active regulation	*D*_*g*_	Reputation premium for the agent seller
*g*	Probability of the agent sellers operating in good faith	*C*_*h*_	The cost of the third-party platforms' careful review
*h*	Probability of third-party platforms taking serious scrutiny	*R*_*h*_	Income of the third-party platforms' operation
*C*_*e*_	The cost of tough government regulation	*S*_*h*_	The premium for third-party platforms' reputation
*R*_*e*_	Social benefits of the government	*D*_*h*_	The loss of the third-party platforms' reputation
*D*_*e*_	Social loss of the government	*F*_*f*_	Amount of government penalties for negatively regulated holders
*C*_*f*_	The cost of active regulation by the holders	*F*_*g*_	Amount of penalties imposed by the Government on trustee-sellers operating in bad faith
*R*_*f*_	Earnings of holders	*F*_*h*_	Amount of penalties imposed by the Government on third-party platforms that conduct perfunctory reviews
*C*_*g*1_	The cost of the honest operation of the agent seller	*I*_*g*1_	compensations of Holders by Fiduciary Sellers Operating in Bad Faith
*C*_*g*2_	The cost of the dishonest operation of the agent seller	*I*_*g*2_	Compensations of third-party platforms by fiduciary sellers operating in bad faith
*R*_*g*_	Earnings of the fiduciary sellers		

### 3.2 Model analytical framework

Based on the above assumptions, this article develops a mixed-strategy game matrix involving the government, holders, trustee-sellers, and third-party platforms, as shown in Table 2.

**Table 2:** Four-party game matrix in the process of pharmaceutical network marketing.

**Choice of strategy**		**Third-party platform**	**Goverment**				
			**Strictly supervise** *e*		**Loosely supervise** 1−*e*	
			**Holder**	**Positive regulation** *f*	**Negative regulation** 1−*f*	**Positive regulation** *f*	**Negative regulation** 1−*f*
Agent seller	Credit management *g*	Strictly review *h*		$\begin{array}{c} R_{e}-C_{e},\\ R_{f}-C_{f},\\ R_{g}+S_{g}-C_{g1},\\ R_{h}+S_{h}-C_{h} \end{array}$	$\begin{array}{c} R_{e}-C_{e},\\ R_{f},\\ R_{g}+S_{g}-C_{g1},\\ R_{h}+S_{h}-C_{h} \end{array}$	$\begin{array}{c} R_{e},\\ R_{f}-C_{f},\\ R_{g}+S_{g}-C_{g1},\\ R_{h}+S_{h}-C_{h} \end{array}$	$\begin{array}{c} R_{\epsilon },\\ R_{f},\\ R_{g}+S_{g}-C_{g1},\\ R_{h}+S_{h}-C_{h} \end{array}$
		Loosely review 1−*h*		$\begin{array}{c} R_{e}-C_{e},\\ R_{f}-C_{f},\\ R_{g}+S_{g}-C_{g1},\\ R_{h} \end{array}$	$\begin{array}{c} R_{e}-C_{e},\\ R_{f},\\ R_{g}+S_{g}-C_{g1},\\ R_{h} \end{array}$	$\begin{array}{c} R_{e},\\ R_{f}-C_{f},\\ R_{g}+S_{g}-C_{g1},\\ R_{h} \end{array}$	$\begin{array}{c} R_{e},\\ R_{f},\\ R_{g}+S_{g}-C_{g1},\\ R_{h} \end{array}$
	Trust-breaking management 1−*g*	Strictly review *h*		$\begin{array}{c} F_{g}-C_{e}-D_{e},\\ R_{f}+I_{g1}-C_{f},\\ R_{g}-C_{g2}-I_{g1}-I_{g2}\\ -D_{g}-F_{g},\\ R_{h}+S_{h}+I_{g2}-C_{h}\\ -D_{h} \end{array}$	$\begin{array}{c} F_{f}+F_{g}-C_{e}-D_{e},\\ R_{f}-F_{f},\\ R_{\text{g}}-C_{\text{g}2}-I_{\text{g}2}\\ -D_{g}-F_{g},\\ R_{h}+S_{h}+I_{g2}-C_{h}\\ -{\text{D}}_{h} \end{array}$	$\begin{array}{c} -D_{e},\\ R_{f}+I_{g1}-C_{f},\\ R_{g}-C_{g2}-I_{g1}\\ -I_{g2}-D_{g},\\ R_{h}+S_{h}+I_{g2}-C_{h}\\ -D_{h} \end{array}$	$\begin{array}{c} -D_{e},\\ R_{f},\\ R_{g}-C_{g2}-I_{g2}-D_{g},\\ R_{h}+S_{h}+I_{g2}-C_{h}\\ -D_{h} \end{array}$
		Loosely review 1−*h*		$\begin{array}{c} F_{g}+F_{h}-C_{e}-D_{e},\\ R_{f}+I_{g1}-C_{f},\\ R_{g}-C_{g2}-I_{g1}\\ -D_{g}-F_{g},\\ R_{h}-D_{h}-F_{h} \end{array}$	$\begin{array}{c} F_{f}+F_{g}+F_{h}-C_{e}\\ -D_{e},\\ R_{f}-F_{f},\\ R_{g}-C_{g2}-D_{g}-F_{g},\\ R_{h}-D_{h}-F_{h} \end{array}$	$\begin{array}{c} -D_{e},\\ R_{f}+I_{g1}-C_{f},\\ R_{g}-C_{g2}-I_{g1}-D_{g},\\ R_{h}-D_{h} \end{array}$	$\begin{array}{c} -D_{e},\\ R_{f},\\ R_{g}-C_{g2}-D_{g},\\ R_{h}-D_{h} \end{array}$

### 3.3 Analysis of the strategic choice stability

#### 3.3.1 Stability analysis of government strategy

The expected return to the government's “tough regulation” strategy is:


$$\begin{array}{l} E_{e}=-C_{e}+g{\text{R}}_{e}+\left(1-g\right)\left[F_{g}-D_{e}+\left(1-h\right)F_{h}+\left(1-f\right)F_{f}\right] \end{array}$$

The expected return to the government's strategy of “lax regulation” is:


$$\begin{array}{l} E_{1-e}=g{\text{R}}_{e}-\left(1-g\right)D_{e} \end{array}$$

The replicated dynamic equations and first-order derivatives of the government strategy choice are:


$$\begin{array}{l} F(e)=de/dt=e(Ee-\bar{E})=e(1-e)(Ee-E1-e)\\ \;=e(1-e)\{-Ce+(1-g)[Fg+(1-h)Fh+(1-f)Ff]\}\\ F^{\prime }(e)=(1-2e)\{-C_{e}+(1-g)[F_{g}+(1-h)F_{h}+(1-f)F_{f}]\} \end{array}$$

According to the stability theorem of the differential equation, when *F*(*e*) = 0⋂*F*′(*e*) < 0, the probability that the government adopts a “tough regulation” strategy is stable.

##### 3.3.1.1 Proposition 1

In case *g*<*g*_1_, the government stabilizes with “tough regulation.” In case *g*>*g*_1_, the government stabilizes with “lax regulation.” In case *g* = *g*_1_, it is impossible to determine its stabilizing strategy. The threshold is


$$\begin{array}{l} g_{1}=1-\frac{C_{e}}{F_{g}+\left(1-h\right)F_{h}+\left(1-f\right)F_{f}} \end{array}$$

Proof: Make *N*(*g*) = −*C*_*e*_+(1−*g*)[*F*_*g*_+(1−*h*)*F*_*h*_+(1−*f*)*F*_*f*_],∂*N*(*g*)/∂*g* < 0, so *N*(*g*) is a reduced function with respect to *g*. When *g*<*g*_1_, *N*(*g*)>0,$F^{\prime }\left(e\right){|}_{e-1}<0$,*F*(*e*)|_*e* = 1_ = 0, then the equilibrium solution *e* = 1 is stable. When *g*>*g*_1_, *N*(*g*) < 0,$F^{\prime }\left(e\right){|}_{e=0}<0$,*F*(*e*)|_*e* = 0_ = 0, then the equilibrium solution *e* = 0 is stable. When *g* = *g*_1_, *N*(*g*) = 0,*F*′(*e*) = 0, its stabilization strategy cannot be determined at this time. The proof is complete.

#### 3.3.2 Stability analysis of the holder's strategy

The holder's expected return from adopting the “active regulation” strategy is:


$$\begin{array}{l} E_{f}=R_{f}-C_{f}+\left(1-g\right)I_{g1} \end{array}$$

The holder's expected return from adopting a “negative regulation” strategy is.


$$\begin{array}{l} E_{1-f}=R_{f}-e\left(1-g\right)F_{f} \end{array}$$

The replicated dynamic equations and first-order derivatives of the holder strategy choice are:


$$\begin{array}{l} F(f)=df/dt=f(E_{f}-\bar{E})=f(1-f)(E_{f}-E_{1-f})\\ \;=f(1-f)[(1-g)(Ig1+eF_{f})-C_{f}]\\ F^{'}(f)=(1-2f)[(1-g)(I_{g1}+eF_{f})-C_{f}] \end{array}$$

According to the stability theorem for differential equations, when *F*(*f*) = 0⋂*F*′(*f*) < 0, the probability of a holder adopting an “active regulation” strategy is stable.

##### 3.3.2.1 Proposition 2

When *g*<*g*_2_, the holders use “active regulation” as a stabilization strategy. When *g*>*g*_2_, holders use “negative regulation” as a stabilization strategy. When *g* = *g*_2_, it is unable to determine its stabilization strategy, the threshold is $g_{2}=1-\frac{C_{f}}{I_{g1}+eF_{f}}$.

Proof: Make *M*(*g*) = (1−*g*)(*I*_*g*1_+*eF*_*f*_)−*C*_*f*_, ∂*M*(*g*)/∂*g* < 0, so *M*(*g*) is a reduced function with respect to *g*. When *g*<*g*_2_, *M*(*g*)>0, then $F^{'}(f){|}_{f-1}<0$, *F*(*f*)|_*f* = 1_ = 0, then the equilibrium solution *f* = 1 is stable. When *g*>*g*_2_, *M*(*g*) < 0, then $F^{'}(f){|}_{f=0}=0$, *F*(*f*)|_*f* = 0_ = 0, then the equilibrium solution *f* = 0 is stable. When *g* = *g*_2_, *M*(*g*) = 0, *F*′(*f*) = 0, its stabilization strategy cannot be determined at this time. The proof is complete.

#### 3.3.3 Stability analysis of the trustee-seller's strategy

The expected return of an agent seller adopting the “honest business” strategy is:


$$\begin{array}{l} E_{g}=R_{g}+S_{g}-C_{g1} \end{array}$$

The expected return to an agent seller adopting the “dishonest business” strategy is:


$$\begin{array}{l} E_{1-g}=R_{g}-C_{g2}-D_{g}-fI_{g1}-hI_{g2}-eF_{g} \end{array}$$

The replicated dynamic equation and first-order derivatives of the agent seller's strategy choice are:


$$\begin{array}{l} F(g)=dg/dt=g(E_{g}-\bar{E})=g(1-g)(E_{g}-E_{1-g})\\ \;=g(1-g)(S_{g}-C_{g1}+C_{g2}+D_{g}+fI_{g1}+hI_{g2}+eF_{g})\\ F^{'}(g)=(1-2g)(S_{g}-C_{g1}+C_{g2}+D_{g}+fI_{g1}+hI_{g2}+eF_{g}) \end{array}$$

According to the stability theorem for differential equations, when *F*(*g*) = 0⋂*F*′(*g*) < 0, the probability that an agent seller will adopt an “honest business” strategy is stable.

##### 3.3.3.1 Proposition 3

When *e*>*e*_0_, *f*>*f*_0_, *h*>*h*_0_, agent sellers use “honest business” as a stabilizing strategy. When *e*<*e*_0_, *f*<*f*_0_, *h*<*h*_0_, agent sellers use “dishonest business” as a stabilization strategy. When *e* = *e*_0_, *f* = *f*_0_, *h* = *h*_0_, it is unable to determine its stabilization strategy, the thresholds are:


$$\begin{array}{l} e_{0}=\frac{C_{g1}-S_{g}-C_{g2}-D_{g}-fI_{g1}-hI_{g2}}{F_{g}},\\ f_{0}=\frac{C_{g1}-S_{g}-C_{g2}-D_{g}-eF_{g}-hI_{g2}}{I_{g1}},\\ h_{0}=\frac{C_{g1}-S_{g}-C_{g2}-D_{g}-eF_{g}-fI_{g1}}{I_{g2}}. \end{array}$$

Proof: Make *N*(*e, f, h*) = *S*_*g*_−*Cg*_1_+*Cg*_2_+*Dg*+*fIg*_1_+*hIg*_2_+*eF*_*g*_, ∂*N*(*e, f, h*)/∂*e*>0, ∂*N*(*e, f, h*)/∂*f*>0, ∂*N*(*e, f, h*)/∂*f*>0, so *N*(*e, f, h*) is an increasing function with respect to *e, f, h*. When *e*>*e*_0_,*f*>*f*_0_,*h*>*h*_0_, then *N*(*e, f, h*)>0,$F^{\prime }\left(g\right){|}_{g=1}<0$, *F*(*g*)|_*g* = 1_ = 0, so the equilibrium solution *g* = 1 is stable. When *e*<*e*_0_, *f*<*f*_0_, *h*<*h*_0_, then*N*(*e, f, h*) < 0, $F^{\prime }\left(g\right){|}_{g=0}<0$, *F*(*g*)|_*g* = 0_ = 0, so the equilibrium solution *g* = 0 is stable. When *e* = *e*_0_,*f* = *f*_0_,*h* = *h*_0_, then *N*(*e, f, h*) = 0, *F*′(*g*) = 0, its stabilization strategy cannot be determined at this time. The proof is complete.

#### 3.3.4 Third-party platform strategy stability analysis

The expected benefits of a third-party platform adopting a “careful vetting” strategy is:


$$\begin{array}{l} E_{h}=R_{h}+S_{h}-C_{h}+\left(1-g\right)\left(I_{g2}-D_{h}\right) \end{array}$$

The expected benefits of the third-party platform's “perfunctory review” strategy is:


$$\begin{array}{l} E_{1-h}=R_{h}-\left(1-g\right)\left(D_{h}+eF_{h}\right) \end{array}$$

The replicated dynamic equations and first-order derivatives of the third-party platform strategy choice are:


$$\begin{array}{l} F\left(h\right)=dh/dt=h\left(E_{h}-Ē\right)=h\left(1-h\right)\left(E_{h}-E_{1-h}\right)\\ =h\left(1-h\right)\left[S_{h}-C_{h}+\left(1-g\right)\left(I_{g2}+eF_{h}\right)\right]\\ F^{\prime }\left(h\right)=\left(1-2h\right)\left[S_{h}-C_{h}+\left(1-g\right)\left(Ig_{2}+eF_{h}\right)\right] \end{array}$$

According to the stability theorem for differential equations, when *F*(*h*) = 0⋂*F*′(*h*) < 0, the probability of a third-party platform adopting a “hard-check” strategy is stable.

##### 3.3.4.1 Proposition 4

When *g*<*g*_3_, third-party platforms are stabilized by “careful reviewing.” When *g*>*g*_3_, third-party platforms use “perfunctory review” as a stabilization strategy. When *g* = *g*_3_, it is unable to determine its stabilization strategy; the threshold is: $g_{3}=1-\frac{C_{h}-S_{h}}{Ig_{2}+eF_{h}}$.

Proof: Make $L\left(g\right)=S_{h}-C_{h}+\left(1-g\right)\left(I_{g^{2}}+eF_{h}\right)$,∂*L*(*g*)/∂*g* < 0, then *L*(*g*) is a reduced function with respect to *g*. When *g*<*g*_3_, then *L*(*g*)>0 $F^{\prime }{\left(h\right)}_{h=1}<0$, *F*(*h*)_*h* = 1_= 0, so the equilibrium solution *h* = 1 is stable. When *g*>*g*_3_, then *L*(*g*) < 0 $F^{\prime }{\left(h\right)}_{h=0}<0$, *F*(*h*)_*h* = 0_= 0, so the equilibrium solution *h* = 0 is stable. When g = g_3_, L(g) = 0, *F*′(*h*) = 0, its stabilization strategy cannot be determined at this time. The proof is complete.

#### 3.3.5 Stability analysis of strategic combination

As stated above, the stability of the 16 pure strategy equilibrium points in the evolutionary game model consisting of four parties, the government, the holder, the agent seller, and the third-party platform, is analyzed through Lyapunov's first method to reveal the conditions and processes for the generation of their stable strategies in the process of pharmaceutical network sales. On this basis, the Jacobian matrix is obtained by replicating the dynamic equations for each game party: $J=\left[\begin{array}{c} \partial F\left(e\right)/\partial e\partial F\left(e\right)/\partial f\partial F\left(e\right)/\partial g\partial F\left(e\right)/\partial h\\ \partial F\left(f\right)/\partial e\partial F\left(f\right)/\partial f\partial F\left(f\right)/\partial g\partial F\left(f\right)/\partial h\\ \partial F\left(g\right)/\partial e\partial F\left(g\right)/\partial f\partial F\left(g\right)/\partial g\partial F\left(g\right)/\partial h\\ \partial F\left(h\right)/\partial e\partial F\left(h\right)/\partial f\partial F\left(h\right)/\partial g\partial F\left(h\right)/\partial h \end{array}\right]$

condition①: F_*f*_-C_*e*_+F_*g*_ < 0, I_*g*1_-C_*f*_ < 0, C_*g*2_-C_*g*1_+I_*g*2_+D_*g*_+S_*g*_ < 0;

condition②: C_*e*_-F_*f*_-F_*g*_ < 0, I_*g*1_-C_*f*_+F_*f*_ < 0, C_*g*2_-C_*g*1_+F_*g*_+I_*g*2_ +D_*g*_+S_*g*_ < 0;

condition③: F_*g*_-C_*e*_ < 0, C_*f*_-I_*g*1_ < 0, C_*g*2_-C_*g*1_+I_*g*1_+I_*g*2_+D_*g*_+S_*g*_ < 0;

condition④: C_*g*1_-C_*g*2_-I_*g*2_- D_*g*_-S_*g*_ < 0;

condition⑤: C_*e*_-F_*g*_ < 0, C_*f*_-F_*f*_-I_*g*1_ < 0, C_*g*2_-C_*g*1_+F_*g*_+I_*g*1_+I_*g*2_ +D_*g*_+S_*g*_ < 0.

When S_*h*_-C_*h*_+ (1 - g)(I_*g*2_+ eF_*h*_) > 0, that is, the asymptotic stability analysis of the equilibrium points of the replicated dynamic system when the stabilization strategy of the third-party platform is a careful review is shown in Table 3.

**Table 3:** Stability analysis of the equilibrium points when carefully reviewed by the third-party platforms.

**Equilibrium point**	**Eigenvalues**	**Sign**	**Stability**
(0,0,0,1)	F_*f*_-C_*e*_+F_*g*_I_*g*1_-C_*f*_, C_*g*2_-C_*g*1_+I_*g*2_+D_*g*_+S_*g*_, C_*h*_−I_*g*2_−*S*_*h*_	(×, ×, ×, −)	ESS when condition ① is satisfied
(1,0,0,1)	C_*e*_-F_*f*_-F_*g*_, I_*g*1_-C_*f*_+F_*f*_, C_*g*2_-C_*g*1_+F_*g*_+I_*g*2_+D_*g*_+S_*g*_C_*h*_−I_*g*2_−*S*_*h*_−*F*_*h*_	(×, ×, ×, −)	ESS when condition ② is satisfied
(0,1,0,1)	F_*g*_-C_*e*_, C_*f*_-I_*g*1_, C_*g*2_-C_*g*1_+I_*g*1_+I_*g*2_+D_*g*_+S_*g*_C_*h*_−I_*g*2_−*S*_*h*_	(×, ×, ×, −)	ESS when condition ③ is satisfied
(0,0,1,1)	-C_*e*_, -C_*f*_, C_*g*1_-C_*g*2_-I_*g*2_−D_*g*_-S_*g*_C_*h*_−*S*_*h*_	(−, −, ×, −)	ESS when condition ④ is satisfied
(1,1,0,1)	C_*e*_-F_*g*_, C_*f*_-F_*f*_-I_*g*1_, C_*g*2_-C_*g*1_+F_*g*_+I_*g*1_+I_*g*2_+D_*g*_+S_*g*_C_*h*_−I_*g*2_−*S*_*h*_−*F*_*h*_		ESS when condition ⑤ is satisfied
(1,0,1,1)	C_*e*_, -C_*f*_, C_*g*1_-C_*g*2_-F_*g*_-I_*g*2_-D_*g*_-S_*g*_, *C*_*h*_−*S*_*h*_	(+, −, ×, −)	Unstable
(0,1,1,1)	-C_*e*_, C_*f*_, C_*g*1_-C_*g*2_-I_*g*1_-I_*g*2_-D_*g*_-S_*g*_, C_*h*_−*S*_*h*_	(−, +, ×, −)	Unstable
(1,1,1,1)	C_*e*_, C_*f*_, C_*g*1_-C_*g*2_-F_*g*_-I_*g*1_-I_*g*2_-D_*g*_-S_*g*_, C_*h*_−*S*_*h*_	(+, +, ×, −)	Unstable

× indicates that the sign is uncertain. If the conditions ,,,, are not met, then they are, respectively, unstable points.

From Table 3, it can be seen that the third-party platform carefully examines the case; if the third-party platform requires an increase in the number of liquidated damages required to be paid by the defaulting business seller and the amount reaches a certain amount, that is, when the condition I_*g*2_+D_*g*_+S_*g*_>C_*g*1_-C_*g*2_ is satisfied, the stabilizing strategy of the agent sellers can be made to be honest business. At this point, the gaming system has only one stable strategy combination (0,0,1,1); when the conditions ①,②,③,⑤ are not satisfied, then (0,0,0,1), (1,0,0,1), (0,1,0,1), (1,1,0,1) are unstable.

condition⑥: F_*f*_-C_*e*_+F_*g*_+F_*h*_ < 0, I_*g*1_-C_*f*_ < 0, C_*g*2_-C_*g*1_+D_*g*_+S_*g*_ < 0;

condition⑦: C_*e*_-F_*f*_-F_*g*_-F_*h*_ < 0, F_*f*_-C_*f*_+I_*g*1_ < 0, C_*g*2_-C_*g*1_+F_*g*_ +D_*g*_+S_*g*_ < 0;

condition⑧: F_*g*_-C_*e*_+F_*h*_ < 0, C_*f*_-I_*g*1_ < 0, C_*g*2_-C_*g*1_+I_*g*1_+D_*g*_+S_*g*_ < 0;

condition⑨: C_*g*1_-C_*g*2_-D_*g*_-S_*g*_ < 0;

condition⑩: C_*e*_-F_*g*_-F_*h*_ < 0, C_*f*_-F_*f*_-I_*g*1_ < 0, C_*g*2_-C_*g*1_+F_*g*_+I_*g*1_ +D_*g*_+S_*g*_ < 0.

When S_*h*_-C_*h*_+ (1 - g)(I_*g*2_+ eF_*h*_) > 0, that is, the asymptotic stability analysis of the equilibrium point of the replicated dynamic system when the stabilization strategy of the third-party platform is perfunctory review is shown in Table 4.

**Table 4:** Stability analysis of the equilibrium points during the perfunctory review of the third-party platforms.

**Equilibrium point**	**Eigenvalues**	**Sign**	**Stability**
(0,0,0,0)	F_*f*_-C_*e*_+F_*g*_+F_*h*_I_*g*1_-C_*f*_, C_*g*2_-C_*g*1_+D_*g*_+S_*g*_, I_*g*2_-C_*h*_+*S*_*h*_	( ×, ×, ×, −)	ESS when condition ⑥ is satisfied
(1,0,0,0)	C_*e*_-F_*f*_-F_*g*_-F_*h*_, F_*f*_-C_*f*_+I_*g*1_, C_*g*2_-C_*g*1_+F_*g*_+D_*g*_+S_*g*_*F*_*h*_−C_*h*_+I_*g*2_+*S*_*h*_	( ×, ×, ×, −)	ESS when condition ⑦ is satisfied
(0,1,0,0)	F_*g*_-C_*e*_+F_*h*_, C_*f*_-I_*g*1_, C_*g*2_-C_*g*1_+I_*g*1_+D_*g*_+S_*g*_I_*g*2_−C_*h*_+*S*_*h*_	( ×, ×, ×, −)	ESS when condition ⑧ is satisfied
(0,0,1,0)	-C_*e*_, -C_*f*_, C_*g*1_-C_*g*2_-D_*g*_-S_*g*_*S*_*h*_-C_*h*_	(−, −, ×, −)	ESS when condition ⑨ is satisfied
(1,1,0,0)	C_*e*_-F_*g*_-F_*h*_, C_*f*_-F_*f*_-I_*g*1_, C_*g*2_-C_*g*1_+F_*g*_+I_*g*1_+D_*g*_+S_*g*_*F*_*h*_−C_*h*_+I_*g*2_+*S*_*h*_	( ×, ×, ×, −)	ESS when condition ⑩ is satisfied
(1,0,1,0)	C_*e*_, -C_*f*_, C_*g*1_-C_*g*2_-F_*g*_-D_*g*_-S_*g*_, *S*_*h*_−*C*_*h*_	(+, −, ×, −)	Unstable
(0,1,1,0)	-C_*e*_, C_*f*_, C_*g*1_-C_*g*2_-I_*g*1_-D_*g*_-S_*g*_, S_*h*_−*C*_*h*_	(−, +, ×, −)	Unstable
(1,1,1,0)	C_*e*_, C_*f*_, C_*g*1_-C_*g*2_-F_*g*_-I_*g*1_-D_*g*_-S_*g*_, S_*h*_−*C*_*h*_	(+, +, ×, −)	Unstable

× indicates that the sign is uncertain. If the conditions ,,,, are not met, then they are, respectively, unstable points.

As shown in Table 4, there are five possible stabilization strategies in the case of a perfunctory review by a third-party platform, where (0,0,1,0) indicates that the government chooses lax regulation, the holder negative regulation, the trustee-seller honest operation, and the third-party platform perfunctory review, and a higher social surplus can be obtained by adopting this strategy. However, there is a discrepancy with the analytical conclusion in Proposition 3, which states that the stabilization strategies of the government, the holder, and the third-party platform do not incentivize trustee-sellers to operate with honesty as a stabilization strategy. Further analysis shows that the following conditions need to be satisfied to obtain a steady state for (0,0,1,0) : C_*g*2_+D_*g*_>C_*g*1_-S_*g*_, that is, the reputation loss that the trustee-seller will face in addition to the operating costs when it operates in bad faith and the reputation premium that it obtains when it operates in good faith is equivalent to reducing some of the costs, and when the former is higher than the latter, the trustee-seller will actively adopt the strategy of operating in good faith. As the reputation loss and reputation premium decrease, the replicated dynamic system will be detached from the optimal state (0,0,1,0), when it reaches a certain level, that is, it satisfies the condition C_*g*2_+D_*g*_ < C_*g*1_-S_*g*_, (0,0,0,0) will be the stabilization point of the replication system. To prevent (0,0,0,0) from becoming a stable equilibrium, at least the condition: F_*f*_+F_*g*_+F_*h*_>C_*e*_ or I_*g*1_>C_*f*_ should be satisfied.

In addition to the three possible stabilization strategies, it can be found that when the third-party platform perfunctory review, such as letting unqualified enterprises carry out drug network operations, not timely stop the illegal behavior of the enterprises stationed, etc., there is no stabilization strategy of the trustee-seller's good faith operation, which will disturb the market order of the Internet drug business, unable to guarantee the quality and safety of medicines, and even jeopardize the life and property of the patients who buy medicines on the Internet. At this time, to avoid the trustee-seller's breach of trust operation to become a stable strategy, the conditions: F_*g*_+D_*g*_+S_*g*_>C_*g*1_-C_*g*2_ should be met, to prevent (1,0,0,0) from becoming ESS. The holder can also increase the amount of liquidated damages to be paid by the agent seller for operating in bad faith so that I_*g*1_+D_*g*_+S_*g*_>C_*g*1_-C_*g*2_, to avoid (0,1,0,0) becoming ESS. Condition ⑩ will not be satisfied, i.e., (1,1,0,0) is the point of instability when one or both of the government and the holder increase the amount of punishment for defaulting business sellers to a certain effectiveness.

#### 3.3.6 Results

It follows from Proposition 1 that if the probability of trustee-sellers operating in good faith decreases, the government's stabilization strategy will change from lax regulation to severe regulation. Therefore, when the government realizes that the probability of trustee-sellers operating in bad faith is higher in drug online sales activities, it will take measures to strengthen the regulation to safeguard the drug online trading environment as well as the safety of the public's life and property.

It follows from Proposition 2 that if the probability of trustee-sellers operating in good faith decreases, the holder's stabilization strategy will change from negative to positive regulation. Under the MAH system, when the holder realizes that the probability of trustee-sellers operating in bad faith during online transactions is high, it will take measures to safeguard its own interests. Therefore, governmental departments can mobilize their active regulation by emphasizing the holder's main responsibility for security and increasing the punishment for the holder's inaction.

It follows from Proposition 3 that the robustness of agent sellers' strategies will shift from integrity to bad faith under the increased probability that the government adopts severe regulation, the holder chooses to regulate aggressively, and the third-party platform conducts careful scrutiny. Therefore, under the MAH system, when trustee producers conduct online drug operations, they will actively comply with the market rules and maintain an honest business environment to avoid penalties in many aspects.

It follows from Proposition 4 that if the probability of agent sellers operating in good faith decreases, the stabilization strategy of the third-party platform will change from a perfunctory review to a serious review. In the pharmaceutical online trading environment, if the probability of agent sellers operating in bad faith is high, the third-party platform will warn and penalize the bad faith enterprises for the consideration of the overall interests of the platform. Therefore, government departments can increase the number of platform spot checks, emphasize the platform's review obligations, and implement other measures to urge the platform to conduct audits and inspections of pharmaceutical network operators.

It follows from Table 3 that careful scrutiny by third-party platforms is of great significance in maintaining the quality and safety of medicines on the Internet. Strengthening government penalties for sellers who operate in breach of trust, holders of passive supervision, and third-party platforms that conduct perfunctory reviews is a key initiative to ensure the quality and safety of medicines.

It follows from Table 4 that, to avoid the undesirable situation, the regulatory role of the government and holders should be fully utilized. The government needs to increase the amount of penalties for sellers, holders, and third-party platforms to reduce the cost of severe regulation, or holders to increase the amount of default of agent sellers, and to reduce the cost of active regulation is also one of the most important measures to avoid sellers operating in bad faith. The government should strengthen the penalty for the breach of trust operation of the pharmaceutical network sales enterprises, and the amount of punishment needs to reach a certain effectiveness. The holder should also raise the liquidated damages amount that must be paid by the agent seller for operating in bad faith.

These results above are logically derived from the model and may overstate the predictability of regulatory outcomes. Therefore, factors that may regulate or undermine the model's predictions are to be considered. For example, these results need to take into account the situation where the trustee-sellers have used certain means to conceal their bad faith operations and dishonest behavior. Moreover, in highly corrupt markets, firms may avoid penalties by bribing regulators or shifting risk rather than changing behavior. The prevalence of such behaviors may offset the deterrent effect of penalties.

## 4 Simulation analysis

To more intuitively show the influence of the changes of key factors in the replicated dynamic system on the evolution process and the evolution results of the multi-party game, this study, on the basis of field research and relevant literature, referenced the parameter settings of Junmei Rong (26). Initially, the parameters are assigned with the corresponding proportion of numerical values, and numerical simulation is conducted by using *MATLAB 2022b* to portray the evolution trajectories of the game subjects in the above model by changing the parameters.

Assume that the cost of strict government regulation *C*_*e*_ = 15, the social gain *R*_*e*_ = 70, the loss to society *D*_*e*_ = 20, the fines to the holder, the seller, and the third-party platform are *F*_*f*_ = 20,*F*_*g*_ = 40,*F*_*h*_ = 20, the cost to the holder of active regulation *C*_*f*_ = 25, and the gain to the holder *R*_*f*_ = 150. The cost reduced by the agent seller's dishonest operation C_*g*1_-C_*g*2_ = 30 and the earning is: *R*_*g*_ = 250; if the dishonest operation is discovered, it is necessary to pay liquidated damages I_*g*1_ = I_*g*2_ = 10. Third-party platform revenue *R*_*h*_ = 100, the cost of careful scrutiny *C*_*h*_ = 10, and the reputation premium *S*_*h*_ = 12. At this point, the strategy choice of the third-party platform is stable and subject to careful scrutiny.

### 4.1 The impact of the agent seller's reputational premium or loss

**Figure 2:**
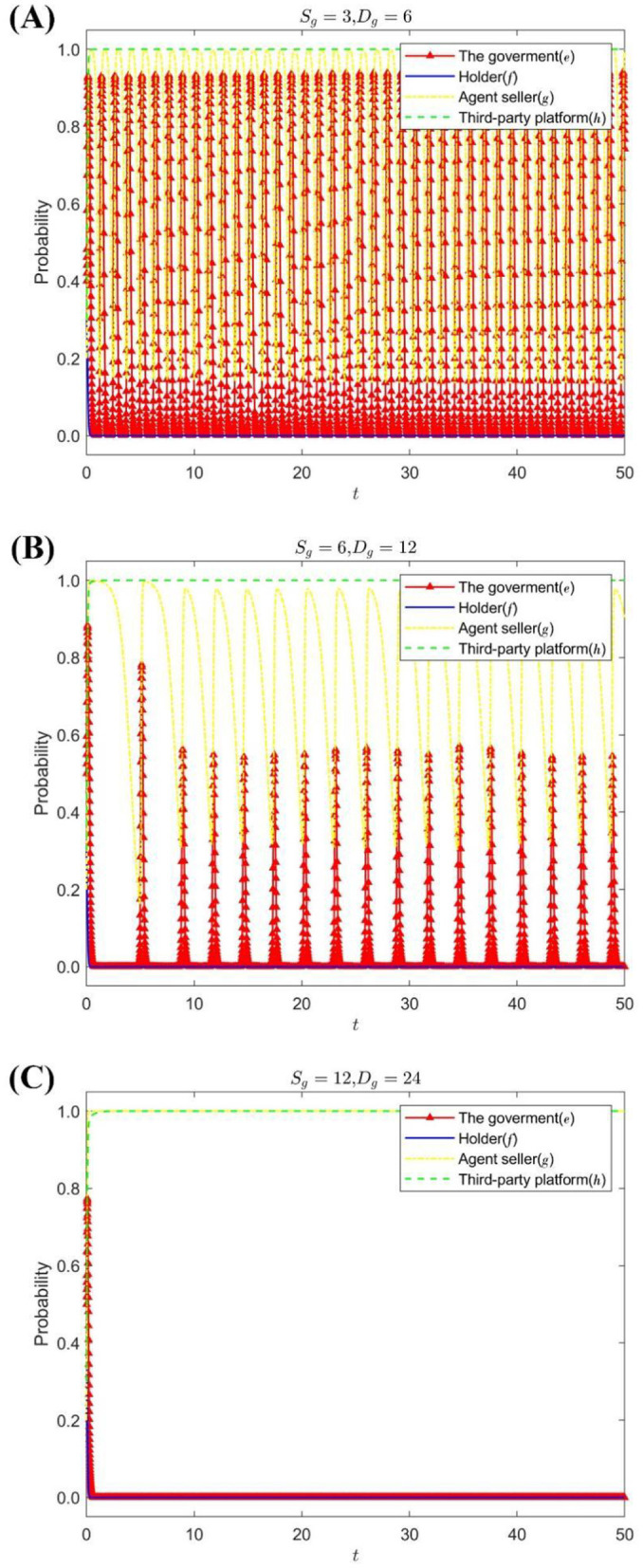
The impact of *S*_*g*_, *D*_*g*_ on the evolution of the strategies of each subject. **(A)** When *S*_*g*_ = 3, *D*_*g*_ = 6. **(B)** When *S*_*g*_ = 6, *D*_*g*_ = 12. **(C)** When *S*_*g*_ = 12, *D*_*g*_ = 24.

A premium or loss of the seller's reputation is set up *S*_*g*_ = {3, 6, 12}, *D*_*g*_ = {6, 12, 24}; Figure 2 shows the strategy evolution process and results of the quadripartite game.

Figure 2 shows that the increase of the reputation premium or loss of the agent seller is helpful for the agent seller to choose a stable strategy of honest management, and the holder and the government choose passive supervision and lax supervision as the stable strategy, respectively. The lower the reputation premium or the less reputational loss of the consignee, the more frequently the consignment seller's strategy changes, the greater the probability fluctuation, and the higher the probability of choosing the strategy of dishonest operation. At the same time, the emergence of dishonest operations of sellers will increase the probability of strict government supervision.

### 4.2 Effect of the amount of default

**Figure 3:**
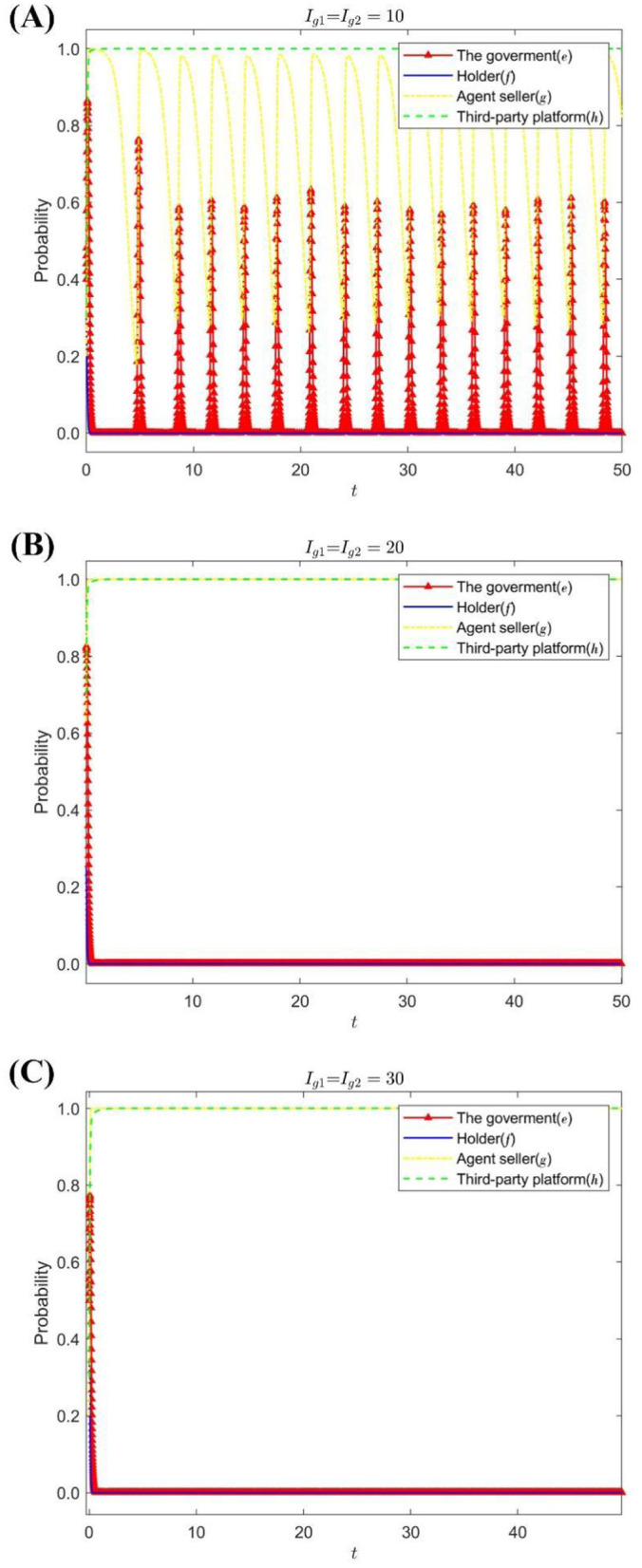
The impact of *I*_*g*1_, *I*_*g*2_ on the evolution of the strategies of each subject. **(A)** When *I*_*g*1_ = *I*_*g*2_ = 10. **(B)** When *I*_*g*1_ = *I*_*g*2_ = 20. **(C)** When *I*_*g*1_ = *I*_*g*2_ = 30.

Under the condition that the increase and the loss of the seller's reputation value is *S*_*g*_ = 6, *D*_*g*_ = 12, change the holder's and third-party platform's requirements for the dishonest business seller to pay the amount of liquidated damages: *I*_*g*1_ = *I*_*g*2_ = {10, 20, 30}. Figure 3 shows the strategy evolution process and results of the quadripartite game.

Figure 3 shows that when the default amount demanded by the holder and the third-party platform for the dishonest seller is small, the speculation of the agent seller will continue to occur, and the government's regulatory strategy will be adjusted accordingly. With the increase in the amount of default required to be paid, the holders adopted passive supervision, and the third-party platform chose to carefully review as a stabilization strategy. After the strategy choice of the agent sellers tended to operate in good faith, the government's stabilization strategy changed to lax supervision.

### 4.3 The impact of the reputation premium of third-party platforms

**Figure 4:**
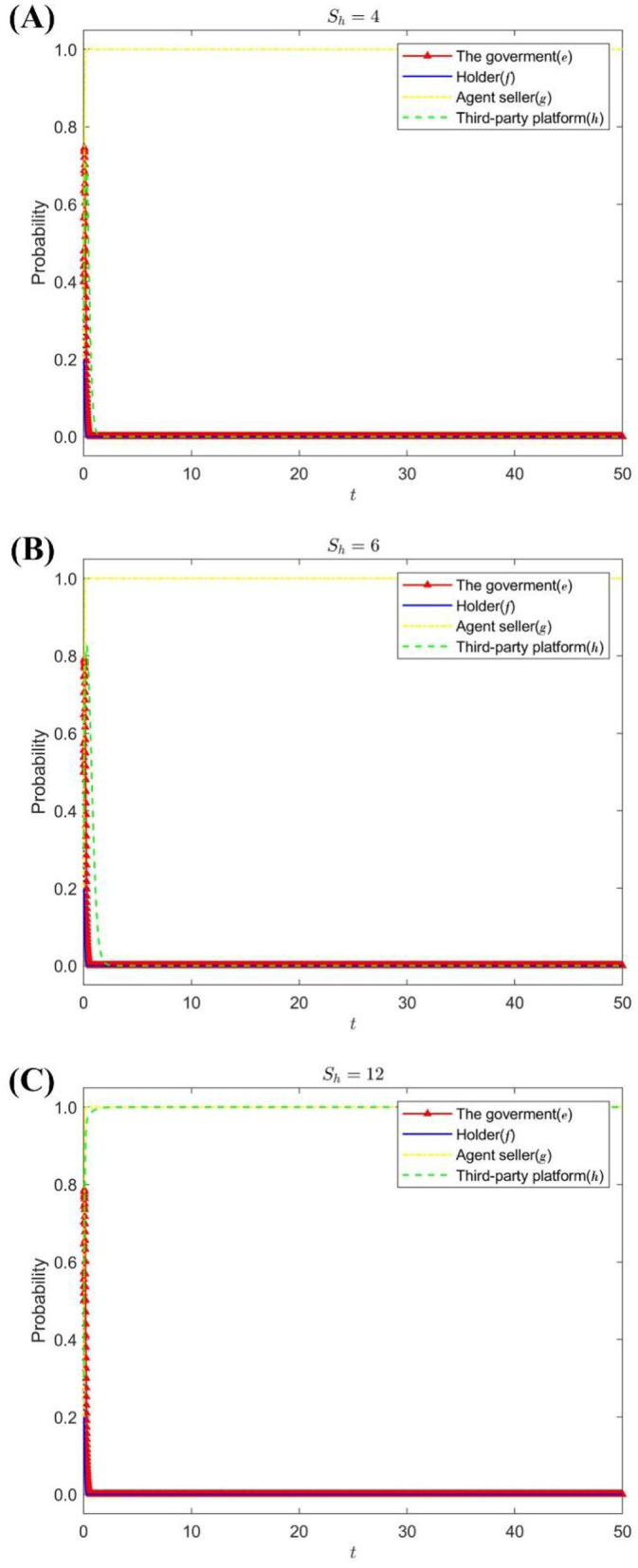
The impact of *S*_*h*_ on the evolution of the strategies of each subject. **(A)** When *S*_*h*_ = 4. **(B)** When *S*_*h*_ = 6. **(C)** When *S*_*h*_ = 12.

Under the condition that the increase of the seller's reputation value is *S*_*g*_ = 12 and the loss of the seller's value is *D*_*g*_ = 24, change the reputation premium of third-party platforms *S*_*h*_ = {4, 6, 12}. Figure 4 shows the strategy evolution process and results of the four parties' game subjects.

Figure 4 shows that when the stable strategy of the agent seller is to operate in good faith and the reputation premium of the third-party platform is low, the strategy choices of the government, the holder, and the third-party platform tend to be lax supervision, passive supervision, and perfunctory review, respectively. As the reputation premium of third-party platforms increases, it will change its stabilization strategy to serious scrutiny to expand the influence of the platform, attract more drug online sales companies to settle in, maintain the positive interaction of consumers, and increase consumer dependence (27), and additional benefits can be obtained.

### 4.4 The impact of government regulatory mechanisms

To verify the effectiveness and feasibility of the government supervision mechanism in the process of drug quality supervision, the parameters of the following arrays are assigned.

Array 1:


$$\begin{array}{l} {\text{C}}_{e}\text{=15,}{\text{C}}_{f}=25,{\text{C}}_{g1}=30,{\text{C}}_{g2}=0,{\text{C}}_{h}\text{=20,}{\text{F}}_{f}=0,{\text{F}}_{g}\text{=10,}{\text{F}}_{h}\text{=0,}\\ {\text{I}}_{g1}\text{=10,}{\text{I}}_{g2}\text{=10,}{\text{S}}_{h}\text{=6,}{\text{S}}_{g}\text{=6,}{\text{D}}_{g}\text{=12} \end{array}$$

Satisfying condition①, the strategy combinations are (lax supervision, negative supervision, operating in bad faith, and perfunctory examination).

Array 2:


$$\begin{array}{l} {\text{C}}_{e}\text{=5,}{\text{C}}_{f}=25,{\text{C}}_{g1}=30,{\text{C}}_{g2}=0,{\text{C}}_{h}\text{=20,}{\text{F}}_{f}=0,{\text{F}}_{g}\text{=10,}{\text{F}}_{h}\text{=0,}\\ {\text{I}}_{g1}\text{=10,}{\text{I}}_{g2}\text{=10,}{\text{S}}_{h}\text{=6,}{\text{S}}_{g}\text{=6,}{\text{D}}_{g}\text{=12} \end{array}$$

Satisfying condition②, the strategy combinations are (strict supervision, passive supervision, dishonest operation, and perfunctory review).

Array 3:


$$\begin{array}{l} {\text{C}}_{e}\text{=5,}{\text{C}}_{f}=25,{\text{C}}_{g1}=30,{\text{C}}_{g2}=0,{\text{C}}_{h}\text{=20,}{\text{F}}_{f}=0,{\text{F}}_{g}\text{=10,}{\text{F}}_{h}\text{=0,}\\ {\text{I}}_{g1}\text{=10,}{\text{I}}_{g2}\text{=10,}{\text{S}}_{h}\text{=6,}{\text{S}}_{g}\text{=12,}{\text{D}}_{g}\text{=24} \end{array}$$

Evolution of strategy portfolios can be mixed and unstable.

Array 4:


$$\begin{array}{l} {\text{C}}_{e}\text{=15,}{\text{C}}_{f}=25,{\text{C}}_{g1}=30,{\text{C}}_{g2}=0,{\text{C}}_{h}\text{=20,}{\text{F}}_{f}=0,{\text{F}}_{g}\text{=10,}{\text{F}}_{h}\text{=0,}\\ {\text{I}}_{g1}\text{=10,}{\text{I}}_{g2}\text{=10,}{\text{S}}_{h}\text{=6,}{\text{S}}_{g}\text{=12,}{\text{D}}_{g}\text{=24} \end{array}$$

Satisfying condition④, the strategy combinations are (lax regulation, negative regulation, honest business, and perfunctory review).

**Figure 5:**
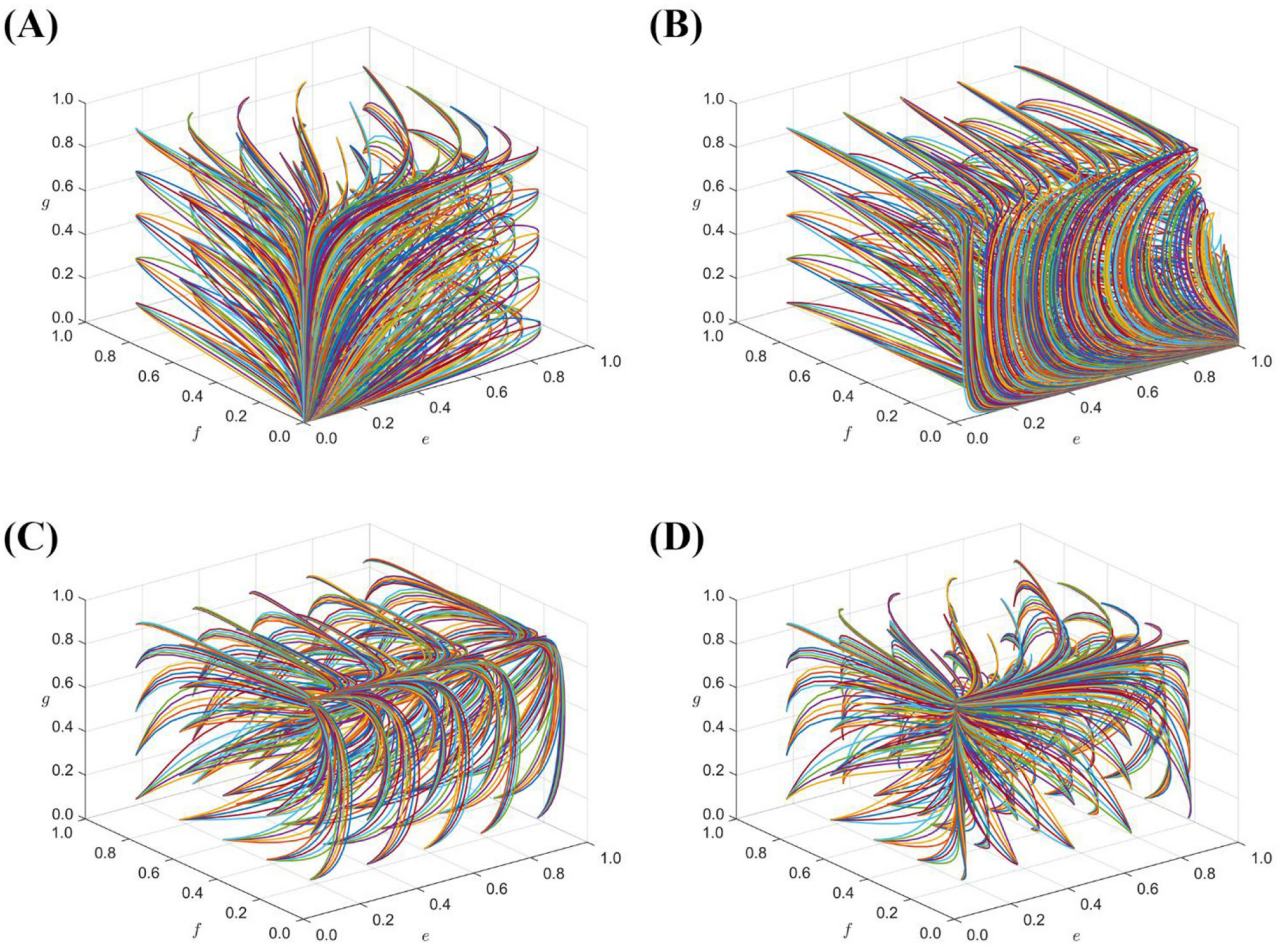
**(A)** is the result of evolving array 1 50 times; **(B)** is the result of evolving array 2 50 times; **(C)** is the result of evolving array 3 50 times; **(D)** is the result of evolving array 4 50 times.

The above four sets of values evolved 50 times over time from different initial strategy combinations to analyze the impact of the government regulatory mechanism in different situations, and the evolution results are shown in Figure 5.

Figure 5A shows that there exists only one stabilization point (0,0,0,0) for the system when conditions, C_*g*2_+D_*g*_ < C_*g*1_-S_*g*_, C_*e*_>F_*f*_+F_*g*_+F_*h*_ C_*f*_>I_*g*1_, C_*h*_>I_*g*2_+S_*h*_ are satisfied, which also implies that the combination of system stabilization strategies is (lax regulation, negative regulation, bad faith operation, and perfunctory review). Figure 5B shows that, based on array 1, reducing the cost of government supervision, when, C_*e*_ < F_*f*_+F_*g*_+F_*h*_ the system will stabilize at the evolutionary stability point (1,0,0,0). At this point, the portfolio of stabilization strategies shifts to (strict supervision, passive supervision, dishonest operation, and perfunctory review). When, C_*g*2_+D_*g*_>C_*g*1_-S_*g*_ Figure 5C shows that, if the cost of government regulation satisfies C_*e*_ < F_*f*_+F_*g*_+F_*h*_, then there is no stabilization point in the system. Figure 5D shows that, when the cost of government regulation is too high, the government tends to choose the stabilization strategy of lax regulation, and there is only one stabilization point (0,0,1,0) in the system. The integrity of the seller's business relies solely on the social level of its reputation, when the seller's breach of trust in business behavior will cause serious impact on public life and health and social harmony and stability. Therefore, to avoid this situation, government departments must increase penalties and reduce regulatory costs. At the same time, it is necessary to establish a market credit and feedback mechanism to improve the ability of sellers to consciously operate in good faith (28), increase the regulatory channels for holders and third-party platforms, effectively safeguard the legitimate rights and interests of consumers, and reduce the pressure on government supervision.

By comparing the MATLAB simulation analysis, it can be seen that this is consistent and effective with the conclusions of the strategy stability analysis of all parties above. It has a certain guiding significance for the quality supervision of online drugs under the MAH system.

## 5 Discussion

This study investigates the quality supervision mechanism of online drug sales under the MAH system from a multi-party game perspective. It develops a hierarchical four-party dynamic evolution game model for online drug sales involving the “government-holder-trusted seller-third-party platform,” solves the eigenvalues by replicating the dynamic system equations, constructs the Jacobi matrix through linkage, and subsequently conducts a numerical analysis to evaluate the role mechanisms of each party and various influencing factors in the model. Using Lyapunov's first method, we determine the evolutionary stable strategy (ESS) of the model, analyze the mechanisms of each game party and the various influencing factors within the model, validate the model through numerical simulation, and propose the following conclusions:

**First, the strategy choices of different game subjects are closely related to their respective strategy costs**. The decrease in the reduced cost of dishonest operation will increase the probability that agent sellers choose to operate in good faith. When the costs increase from low to high for strict and active regulation, the government, the holder, and the third-party platform shift from enforcing strict and active regulation to negative regulation.

**Second, the government's regulatory mechanism and the level of incentives and penalties guide the strategic choices of the game players**. The government is likely to gradually evolve toward lax regulation due to excessive expenditure on rewards. When the government's punishment is too low, all parties choose speculative behavior to achieve greater gains, and the probability of strict government regulation will decrease.

**Third, the participation of holders and third-party platforms is important for ensuring the quality of medication**. Increasing the amount of liquidated damages that must be paid by untrustworthy agent sellers operating in bad faith when actively regulated by holders or those carefully scrutinized by third-party platforms, respectively, will, to a certain extent, make them choose to operate with integrity.

**Fourth, there are cases of ineffective regulation by one or more parties when multiple parties are involved in the regulatory review process**. Careful review by the third-party platform can reduce the number of non-compliant agent sellers entering the market for Internet drug business, and the probability of severe government regulation and active regulation by the holders will be reduced.

**Finally, reputation premium or loss has an effect on the agent seller as well as the third-party platform**. When the trustee-seller's reputation premium or loss is small, the frequency of changes in the agent sellers' strategy increases, and an increase in the reputation premium or loss accelerates the sellers' honesty in business.

## 6 Suggestions

The establishment of the Marketing Authorization Holder (MAH) system and the expansion of online drug businesses have increased the number of entities engaged in drug quality regulation. Effectively controlling the quality of online drugs and implementing a multi-party collaborative regulatory strategy is a shared challenge (29). Based on the study findings, the following policy implications can be suggested:

(1) The first step is to improve the regulatory mechanism, focusing on enhancing transparency in the pharmaceutical industry and increasing government supervision efficiency. This involves encouraging multiple parties to participate in oversight and gaining a comprehensive understanding of market supervision processes. Holders should prioritize using agreements to constrain the actions of authorized sellers and regulate the conduct of manufacturers and sellers. Moreover, enhancing the professional skills of relevant personnel and emphasizing accountability for each stage of the online medicine sales process is crucial to swiftly addressing regulatory violations (30).

(2) It is imperative to establish a fair system of rewards and penalties. Provide specific policy backing to holders, sellers, and third-party platforms demonstrating a strong commitment to social responsibility (31), while simultaneously raising penalties for issues related to drug quality, thereby escalating the consequences of non-compliance for all involved. This will foster a conducive business environment.

(3) The development of a compensation mechanism for information sharing can foster the establishment of a robust co-governance regulatory framework. First, the government should formulate relevant policies and clarify the rules and standards for information sharing. In addition, reward units that make significant contributions to information sharing and penalize subjects that fail to fulfill their information sharing obligations accordingly. Active engagement in sharing information on medicine quality will be emphasized in this system, reducing information discrepancies and delays (32).

(4) The deregulation of prices and the enhancement of feedback channels are imperative for market regulation effectiveness. Emphasis should be placed on developing new media platforms to oversee the dissemination of information concerning drug quality, educate the public on safe medication use, underscore the significance of patient feedback (33), and uphold patients' rights to partake in regulatory procedures.

Overall, our research conclusions have potential applicability within the international pharmaceutical regulatory framework. For instance, both EU countries and the United States possess relatively well-established regulatory systems for drug oversight, and enhancing transparency and improving regulatory mechanisms align with their existing core regulatory principles (34). Establishing clear systems of rewards and penalties contributes to the standardization of the overall pharmaceutical market and the establishment of consistent operational norms. In this era of promoting information transparency and sharing, developing a compensation mechanism for information sharing is also crucial. Against the backdrop of increasing attention to global public health and drug safety, these recommendations can effectively facilitate international cooperation and experience sharing, providing important support for advancing drug quality regulation.

Several limitations should be acknowledged in this study: The focus is on the quality regulation of pharmaceuticals in online sales, considering asymmetric information and limited rationality. However, drugs go through multiple stages, including R&D, procurement, production, testing, storage, distribution, and sales, before reaching consumers. It is suggested that this could be followed by studies on the impact of the various parties in the pharmaceutical supply chains and the complex interactions between these stages are essential for comprehensive pharmaceutical quality management. Despite the considerable growth in online sales channels, offline sales remain the primary focus of the pharmaceutical industry (35). Therefore, the competitive relationship between online and offline drug sales channels should be the entry point in introducing the concept of the drug supply chain. Future research should examine the mechanisms through which each element in the entire process of drug quality supervision affects drug quality, aiming to further improve the drug quality supervision mechanism.

## Data Availability

The original contributions presented in the study are included in the article/Supplementary material, further inquiries can be directed to the corresponding authors.
