# Supplementary material for: Research on the quality supervision strategy of drugs sold on the internet under the MAH system
Source: Front Public Health. 2025 Apr 9;13:1457340. doi: 10.3389/fpubh.2025.1457340 (PMC12014731; doi:10.3389/fpubh.2025.1457340)
Supplement: Supplementary file 1 [file Data_Sheet_1.docx]

# Supplementary Data

Quadratic evolutionary game and simulation analysis code

| 1. Solving replicated dynamic equations and eigenvalues of the Jacobi matrix |
| --- |
| syms e f g h Re Rf Rh Rg Ce Cf Ff Fg Fh Ig1 Ig2 Cg1 Cg2 Ch Dh Sh De Dg Sg;  %Government expectations and replication of dynamic equations  V11=(Re-Ce)*f*g*h+(Re-Ce)*(1-f)*g*h+(Re-Ce)*f*g*(1-h)+(Re-Ce)*(1-f)*g*(1-h)+(Fg-Ce-De)*f*(1-g)*h+(Ff+Fg-Ce-De)*(1-f)*(1-g)*h+(Fg+Fh-Ce-De)*f*(1-g)*(1-h)+(Ff+Fg+Fh-Ce-De)*(1-f)*(1-g)*(1-h);  V12=Re*f*g*h+Re*(1-f)*g*h+Re*f*g*(1-h)+Re*(1-f)*g*(1-h)-De*f*(1-g)*h-De*(1-f)*(1-g)*h-De*f*(1-g)*(1-h)-De*(1-f)*(1-g)*(1-h);  V1=simplify(e*V11+(1-e)*V12);  fe=simplify(e*(V11-V1));  %Holder's expectation and replication dynamic equations  V21=(Rf-Cf)*e*g*h+(Rf-Cf)*e*g*(1-h)+(Rf+Ig1-Cf)*e*(1-g)*h+(Rf+Ig1-Cf)*e*(1-g)*(1-h)+(Rf-Cf)*(1-e)*g*h+(Rf-Cf)*(1-e)*g*(1-h)+(Rf+Ig1-Cf)*(1-e)*(1-g)*h+(Rf+Ig1-Cf)*(1-e)*(1-g)*(1-h);  V22=Rf*e*g*h+Rf*e*g*(1-h)+(Rf-Ff)*e*(1-g)*h+(Rf-Ff)*e*(1-g)*(1-h)+Rf*(1-e)*g*h+Rf*(1-e)*g*(1-h)+Rf*(1-e)*(1-g)*h+Rf*(1-e)*(1-g)*(1-h);  V2=simplify(f*V21+(1-f)*V22);  ff=simplify(f*(V21-V2));  %Expectation and replication dynamics equations for entrusted sellers  V31=(Rg+Sg-Cg1)*e*f*h+(Rg+Sg-Cg1)*e*(1-f)*h+(Rg+Sg-Cg1)*(1-e)*f*h+(Rg+Sg-Cg1)*(1-e)*(1-f)*h+(Rg+Sg-Cg1)*e*f*(1-h)+(Rg+Sg-Cg1)*e*(1-f)*(1-h)+(Rg+Sg-Cg1)*(1-e)*f*(1-h)+(Rg+Sg-Cg1)*(1-e)*(1-f)*(1-h);  V32=(Rg-Cg2-Ig1-Ig2-Fg-Dg)*e*f*h+(Rg-Cg2-Ig2-Fg-Dg)*e*(1-f)*h+(Rg-Cg2-Ig1-Ig2-Dg)*(1-e)*f*h+(Rg-Cg2-Ig2-Dg)*(1-e)*(1-f)*h+(Rg-Cg2-Ig1-Fg-Dg)*e*f*(1-h)+(Rg-Cg2-Fg-Dg)*e*(1-f)*(1-h)+(Rg-Cg2-Ig1-Dg)*(1-e)*f*(1-h)+(Rg-Cg2-Dg)*(1-e)*(1-f)*(1-h);  V3=simplify(g*V31+(1-g)*V32);  fg=simplify(g*(V31-V3));  %Expectations of third-party platforms and replication of dynamic equations  V41=(Rh+Sh-Ch)*e*f*g+(Rh+Sh-Ch)*e*(1-f)*g+(Rh+Sh-Ch)*(1-e)*f*g+(Rh+Sh-Ch)*(1-e)*(1-f)*g+(Rh+Sh+Ig2-Ch-Dh)*e*f*(1-g)+(Rh+Sh+Ig2-Ch-Dh)*e*(1-f)*(1-g)+(Rh+Sh+Ig2-Ch-Dh)*(1-e)*f*(1-g)+(Rh+Sh+Ig2-Ch-Dh)*(1-e)*(1-f)*(1-g);  V42=Rh*e*f*g+Rh*e*(1-f)*g+Rh*(1-e)*f*g+Rh*(1-e)*(1-f)*g+(Rh-Fh-Dh)*e*(1-f)*(1-g)+(Rh-Dh)*(1-e)*(1-f)*(1-g)+(Rh-Fh-Dh)*e*f*(1-g)+(Rh-Dh)*(1-e)*f*(1-g);  V4=simplify(h*V41+(1-h)*V42);  fh=simplify(h*(V41-V4));  %Find the equilibrium point and its eigenvalues  disp(['Jacobi matrix']);  A=[diff(fe,e) diff(fe,f) diff(fe,g) diff(fe,h);  diff(ff,e) diff(ff,f) diff(ff,g) diff(ff,h);  diff(fg,e) diff(fg,f) diff(fg,g) diff(fg,h);  diff(fh,e) diff(fh,f) diff(fh,g) diff(fh,h)]  equ=[fe==0,ff==0,fg==0,fh==0];  answ=solve(equ,[e,f,g,h]);  disp(['Equilibrium point：']);A1=[answ.e,answ.f,answ.g,answ.h]  disp(['Number of equalization points：']);length(answ.e)  xz=[];  for j=9:24  disp(['The' num2str(j) ' equilibrium point：']);  A1(j,:)  disp(['The' num2str(j) ' Matrix after equilibrium points are brought into the Jacobi matrix：']);  e1=A1(j,1);f1=A1(j,2);g1=A1(j,3);h1=A1(j,4);  B=subs(A,[e f g h],[e1 f1 g1 h1])  disp(['The' num2str(j) ' Eigenvalues of the equilibrium points brought to the Jacobi matrix：']);  [V,R] = eig(B);  B1=R(1,1)  B2=R(2,2)  B3=R(3,3)  B4=R(4,4)  xz=[xz;B1,B2,B3,B4];  end  disp([' The set of eigenvalues in the first column：']);xz(:,1)  disp([' The set of eigenvalues in the second column：']);xz(:,2)  disp([' The set of eigenvalues in the third column：']);xz(:,3)  disp([' The set of eigenvalues in the fourth column：']);xz(:,4) |

| **2.** Evolutionary process diagram |
| --- |
| **Script 1 (Quadratic game function definition)** |
| function dydt=FP(t,y,Ce,Cf,Cg1,Cg2,Ch,Ff,Fg,Fh,Ig1,Ig2,Sh,Sg,Dg)  dydt=zeros(4,1);  dydt(1)=y(1)*(1-y(1))*(-Ce+(1-y(3))*(Fg+(1-y(4))*Fh+(1-y(2))*Ff));  dydt(2)=y(2)*(1-y(2))*((1-y(3))*(Ig1+y(1)*Ff)-Cf);  dydt(3)=y(3)*(1-y(3))*(-Cg1+Cg2+Sg+Dg+y(2)*Ig1+y(4)*Ig2+y(1)*Fg);  dydt(4)=y(4)*(1-y(4))*(Sh-Ch+(1-y(3))*Ig2+y(1)*Fh);  end |
| **Script 2 (Agent seller's reputation premium or loss)** |
| %Sg/Dg large picture  figure(1)  % subplot 1  clc;clear;  Ce=15,Cf=25,Cg1=30,Cg2=0,Ch=10,Ff=20,Fg=40,Fh=20,Ig2=10,Ig1=10,Sh=12,Sg=3,Dg=6;  set(0,'defaultfigurecolor','w')  [t,y]=ode45(@(t,y) FP(t,y,Ce,Cf,Cg1,Cg2,Ch,Ff,Fg,Fh,Ig1,Ig2,Sh,Sg,Dg),[0,50],[0.4,0.3,0.2,0.3]);  points=1:1:length(t);  plot(t,y(:,1),'r^-','linewidth',1,'markersize',3,'markerfacecolor','r','markerindices',points);  hold on  plot(t,y(:,2),'b-','linewidth',1);  hold on  plot(t,y(:,3),'y-.','linewidth',1);  hold on  plot(t,y(:,4),'g--','linewidth',1);  hold on  set(gca,'XTick',[0:10:50],'YTick',[0.0:0.2:1.0])  set(gca,'YTickLabel',num2str(get(gca,'YTick')','%.1f'));  axis([0 50 -0.05 1.05])  xlabel('$t$','interpreter','latex');  ylabel('Probability');  zhuti=title('$S_{g}=3$,$D_{g}=6$');  set(zhuti,'interpreter','latex')  legend('The goverment({\it\fontname{Bodoni MT}e})','Holder({\it\fontname{Bodoni MT}f})','Agent seller({\it\fontname{Bodoni MT}g})','Third-party platform({\it\fontname{Bodoni MT}h})');  %subplot 2  clc;clear;  Ce=15,Cf=25,Cg1=30,Cg2=0,Ch=10,Ff=20,Fg=40,Fh=20,Ig2=10,Ig1=10,Sh=12,Sg=6,Dg=12;  figure(2)  set(0,'defaultfigurecolor','w')  [t,y]=ode45(@(t,y) FP(t,y,Ce,Cf,Cg1,Cg2,Ch,Ff,Fg,Fh,Ig1,Ig2,Sh,Sg,Dg),[0,50],[0.5,0.3,0.2,0.3]);  points=1:1:length(t);  plot(t,y(:,1),'r^-','linewidth',1,'markersize',3,'markerfacecolor','r','markerindices',points);  hold on  plot(t,y(:,2),'b-','linewidth',1);  hold on  plot(t,y(:,3),'y-.','linewidth',1);  hold on  plot(t,y(:,4),'g--','linewidth',1);  hold on  set(gca,'XTick',[0:10:50],'YTick',[0.0:0.2:1.0])  set(gca,'YTickLabel',num2str(get(gca,'YTick')','%.1f'));  axis([0 50 -0.05 1.05])  xlabel('$t$','interpreter','latex');  ylabel('Probability');  zhuti=title('$S_{g}=6$,$D_{g}=12$');  set(zhuti,'interpreter','latex')  legend('The goverment({\it\fontname{Bodoni MT}e})','Holder({\it\fontname{Bodoni MT}f})','Agent seller({\it\fontname{Bodoni MT}g})','Third-party platform({\it\fontname{Bodoni MT}h})');  %subplot 3  clc;clear;  Ce=15,Cf=25,Cg1=30,Cg2=0,Ch=10,Ff=20,Fg=40,Fh=20,Ig2=10,Ig1=10,Sh=12,Sg=12,Dg=24;  figure(3)  set(0,'defaultfigurecolor','w')  [t,y]=ode45(@(t,y) FP(t,y,Ce,Cf,Cg1,Cg2,Ch,Ff,Fg,Fh,Ig1,Ig2,Sh,Sg,Dg),[0,50],[0.5,0.3,0.2,0.3]);  points=1:1:length(t);  plot(t,y(:,1),'r^-','linewidth',1,'markersize',3,'markerfacecolor','r','markerindices',points);  hold on  plot(t,y(:,2),'b-','linewidth',1);  hold on  plot(t,y(:,3),'y-.','linewidth',1);  hold on  plot(t,y(:,4),'g--','linewidth',1);  hold on  set(gca,'XTick',[0:10:50],'YTick',[0.0:0.2:1.0])  set(gca,'YTickLabel',num2str(get(gca,'YTick')','%.1f'));  axis([0 50 -0.05 1.05])  xlabel('$t$','interpreter','latex');  ylabel('Probability');  zhuti=title('$S_{g}=12$,$D_{g}=24$');  set(zhuti,'interpreter','latex')  legend('The goverment({\it\fontname{Bodoni MT}e})','Holder({\it\fontname{Bodoni MT}f})','Agent seller({\it\fontname{Bodoni MT}g})','Third-party platform({\it\fontname{Bodoni MT}h})'); |
| **Script 3 (** **Effect of the Amount of Default)** |
| %Sg/Dg large picture  figure(1)  % subplot 1  clc;clear;  Ce=15,Cf=25,Cg1=30,Cg2=0,Ch=10,Ff=20,Fg=40,Fh=20,Ig2=10,Ig1=10,Sh=12,Sg=6,Dg=12;  set(0,'defaultfigurecolor','w')  [t,y]=ode45(@(t,y) FP(t,y,Ce,Cf,Cg1,Cg2,Ch,Ff,Fg,Fh,Ig1,Ig2,Sh,Sg,Dg),[0,50],[0.4,0.3,0.2,0.3]);  points=1:1:length(t);  plot(t,y(:,1),'r^-','linewidth',1,'markersize',3,'markerfacecolor','r','markerindices',points);  hold on  plot(t,y(:,2),'b-','linewidth',1);  hold on  plot(t,y(:,3),'y-.','linewidth',1);  hold on  plot(t,y(:,4),'g--','linewidth',1);  hold on  set(gca,'XTick',[0:10:50],'YTick',[0.0:0.2:1.0])  set(gca,'YTickLabel',num2str(get(gca,'YTick')','%.1f'));  axis([0 50 -0.05 1.05])  xlabel('$t$','interpreter','latex');  ylabel('Probability');  zhuti=title('$I_{g1}=10$,$I_{g2}=10$');  set(zhuti,'interpreter','latex')  legend('The goverment({\it\fontname{Bodoni MT}e})','Holder({\it\fontname{Bodoni MT}f})','Agent seller({\it\fontname{Bodoni MT}g})','Third-party platform({\it\fontname{Bodoni MT}h})');  %subplot 2  clc;clear;  Ce=15,Cf=25,Cg1=30,Cg2=0,Ch=10,Ff=20,Fg=40,Fh=20,Ig2=20,Ig1=20,Sh=12,Sg=6,Dg=12;  figure(2)  set(0,'defaultfigurecolor','w')  [t,y]=ode45(@(t,y) FP(t,y,Ce,Cf,Cg1,Cg2,Ch,Ff,Fg,Fh,Ig1,Ig2,Sh,Sg,Dg),[0,50],[0.5,0.3,0.2,0.3]);  points=1:1:length(t);  plot(t,y(:,1),'r^-','linewidth',1,'markersize',3,'markerfacecolor','r','markerindices',points);  hold on  plot(t,y(:,2),'b-','linewidth',1);  hold on  plot(t,y(:,3),'y-.','linewidth',1);  hold on  plot(t,y(:,4),'g--','linewidth',1);  hold on  set(gca,'XTick',[0:10:50],'YTick',[0.0:0.2:1.0])  set(gca,'YTickLabel',num2str(get(gca,'YTick')','%.1f'));  axis([0 50 -0.05 1.05])  xlabel('$t$','interpreter','latex');  ylabel('Probability');  zhuti=title('$I_{g1}=20$,$I_{g2}=20$');  set(zhuti,'interpreter','latex')  legend('The goverment({\it\fontname{Bodoni MT}e})','Holder({\it\fontname{Bodoni MT}f})','Agent seller({\it\fontname{Bodoni MT}g})','Third-party platform({\it\fontname{Bodoni MT}h})');  %subplot 3  clc;clear;  Ce=15,Cf=25,Cg1=30,Cg2=0,Ch=10,Ff=20,Fg=40,Fh=20,Ig2=30,Ig1=30,Sh=12,Sg=6,Dg=12;  figure(3)  set(0,'defaultfigurecolor','w')  [t,y]=ode45(@(t,y) FP(t,y,Ce,Cf,Cg1,Cg2,Ch,Ff,Fg,Fh,Ig1,Ig2,Sh,Sg,Dg),[0,50],[0.5,0.3,0.2,0.3]);  points=1:1:length(t);  plot(t,y(:,1),'r^-','linewidth',1,'markersize',3,'markerfacecolor','r','markerindices',points);  hold on  plot(t,y(:,2),'b-','linewidth',1);  hold on  plot(t,y(:,3),'y-.','linewidth',1);  hold on  plot(t,y(:,4),'g--','linewidth',1);  hold on  set(gca,'XTick',[0:10:50],'YTick',[0.0:0.2:1.0])  set(gca,'YTickLabel',num2str(get(gca,'YTick')','%.1f'));  axis([0 50 -0.05 1.05])  xlabel('$t$','interpreter','latex');  ylabel('Probability');  zhuti=title('$I_{g1}=30$,$I_{g2}=30$');  set(zhuti,'interpreter','latex')  legend('The goverment({\it\fontname{Bodoni MT}e})','Holder({\it\fontname{Bodoni MT}f})','Agent seller({\it\fontname{Bodoni MT}g})','Third-party platform({\it\fontname{Bodoni MT}h})'); |
| **Script 4 (The Impact of the Reputation Premium of Third-party Platforms)** |
| %Sg/Dg large picture  figure(1)  % subplot 1  clc;clear;  Ce=15,Cf=25,Cg1=30,Cg2=0,Ch=10,Ff=20,Fg=40,Fh=20,Ig2=10,Ig1=10,Sh=4,Sg=12,Dg=24;  set(0,'defaultfigurecolor','w')  [t,y]=ode45(@(t,y) FP(t,y,Ce,Cf,Cg1,Cg2,Ch,Ff,Fg,Fh,Ig1,Ig2,Sh,Sg,Dg),[0,50],[0.4,0.3,0.2,0.3]);  points=1:1:length(t);  plot(t,y(:,1),'r^-','linewidth',1,'markersize',3,'markerfacecolor','r','markerindices',points);  hold on  plot(t,y(:,2),'b-','linewidth',1);  hold on  plot(t,y(:,3),'y-.','linewidth',1);  hold on  plot(t,y(:,4),'g--','linewidth',1);  hold on  set(gca,'XTick',[0:10:50],'YTick',[0.0:0.2:1.0])  set(gca,'YTickLabel',num2str(get(gca,'YTick')','%.1f'));  axis([0 50 -0.05 1.05])  xlabel('$t$','interpreter','latex');  ylabel('Probability');  zhuti=title('$S_{h}=4$');  set(zhuti,'interpreter','latex')  legend('The goverment({\it\fontname{Bodoni MT}e})','Holder({\it\fontname{Bodoni MT}f})','Agent seller({\it\fontname{Bodoni MT}g})','Third-party platform({\it\fontname{Bodoni MT}h})');  %subplot 2  clc;clear;  Ce=15,Cf=25,Cg1=30,Cg2=0,Ch=10,Ff=20,Fg=40,Fh=20,Ig2=10,Ig1=10,Sh=6,Sg=12,Dg=24;  figure(2)  set(0,'defaultfigurecolor','w')  [t,y]=ode45(@(t,y) FP(t,y,Ce,Cf,Cg1,Cg2,Ch,Ff,Fg,Fh,Ig1,Ig2,Sh,Sg,Dg),[0,50],[0.5,0.3,0.2,0.3]);  points=1:1:length(t);  plot(t,y(:,1),'r^-','linewidth',1,'markersize',3,'markerfacecolor','r','markerindices',points);  hold on  plot(t,y(:,2),'b-','linewidth',1);  hold on  plot(t,y(:,3),'y-.','linewidth',1);  hold on  plot(t,y(:,4),'g--','linewidth',1);  hold on  set(gca,'XTick',[0:10:50],'YTick',[0.0:0.2:1.0])  set(gca,'YTickLabel',num2str(get(gca,'YTick')','%.1f'));  axis([0 50 -0.05 1.05])  xlabel('$t$','interpreter','latex');  ylabel('Probability');  zhuti=title('$S_{h}=6$');  set(zhuti,'interpreter','latex')  legend('The goverment({\it\fontname{Bodoni MT}e})','Holder({\it\fontname{Bodoni MT}f})','Agent seller({\it\fontname{Bodoni MT}g})','Third-party platform({\it\fontname{Bodoni MT}h})');  %subplot 3  clc;clear;  Ce=15,Cf=25,Cg1=30,Cg2=0,Ch=10,Ff=20,Fg=40,Fh=20,Ig2=10,Ig1=10,Sh=12,Sg=12,Dg=24;  figure(3)  set(0,'defaultfigurecolor','w')  [t,y]=ode45(@(t,y) FP(t,y,Ce,Cf,Cg1,Cg2,Ch,Ff,Fg,Fh,Ig1,Ig2,Sh,Sg,Dg),[0,50],[0.5,0.3,0.2,0.3]);  points=1:1:length(t);  plot(t,y(:,1),'r^-','linewidth',1,'markersize',3,'markerfacecolor','r','markerindices',points);  hold on  plot(t,y(:,2),'b-','linewidth',1);  hold on  plot(t,y(:,3),'y-.','linewidth',1);  hold on  plot(t,y(:,4),'g--','linewidth',1);  hold on  set(gca,'XTick',[0:10:50],'YTick',[0.0:0.2:1.0])  set(gca,'YTickLabel',num2str(get(gca,'YTick')','%.1f'));  axis([0 50 -0.05 1.05])  xlabel('$t$','interpreter','latex');  ylabel('Probability');  zhuti=title('$S_{h}=12$');  set(zhuti,'interpreter','latex')  legend('The goverment({\it\fontname{Bodoni MT}e})','Holder({\it\fontname{Bodoni MT}f})','Agent seller({\it\fontname{Bodoni MT}g})','Third-party platform({\it\fontname{Bodoni MT}h})'); |
| **Script 5 (code for the impact of government regulatory mechanisms)** |
| %Array1  clc;clear;  Ce=15,Cf=25,Cg1=30,Cg2=0,Ch=20,Ff=0,Fg=10,Fh=0,Ig2=10,Ig1=10,Sh=6,Sg=6,Dg=12;  figure(1)  for i=0.1:0.2:1  for j=0.1:0.2:1  for k=0.1:0.2:1  for l=0.1:0.2:1  [t,y]=ode45(@(t,y) FP(t,y,Ce,Cf,Cg1,Cg2,Ch,Ff,Fg,Fh,Ig1,Ig2,Sh,Sg,Dg),[0 50],[i j k l]);  grid on  plot3(y(:,1),y(:,2),y(:,3),'linewidth',1);  set(gca,'XTick',[0:0.2:1],'YTick',[0:0.2:1],'ZTick',[0:0.2:1])  set(gca,'XTickLabel',num2str(get(gca,'XTick')','%.1f'));  set(gca,'YTickLabel',num2str(get(gca,'YTick')','%.1f'));  set(gca,'ZTickLabel',num2str(get(gca,'ZTick')','%.1f'));  hold on  axis([0 1 0 1 0 1])  end  end  end  end  xlabel('$e$','interpreter','latex');  ylabel('$f$','interpreter','latex');  zlabel('$g$','interpreter','latex','Rotation',360)；  %Array2  clc;clear;  Ce=5,Cf=25,Cg1=30,Cg2=0,Ch=20,Ff=0,Fg=10,Fh=0,Ig2=10,Ig1=10,Sh=6,Sg=6,Dg=12;  figure(1)  for i=0.1:0.2:1  for j=0.1:0.2:1  for k=0.1:0.2:1  for l=0.1:0.2:1  [t,y]=ode45(@(t,y) FP(t,y,Ce,Cf,Cg1,Cg2,Ch,Ff,Fg,Fh,Ig1,Ig2,Sh,Sg,Dg),[0 50],[i j k l]);  grid on  plot3(y(:,1),y(:,2),y(:,3),'linewidth',1);  set(gca,'XTick',[0:0.2:1],'YTick',[0:0.2:1],'ZTick',[0:0.2:1])  set(gca,'XTickLabel',num2str(get(gca,'XTick')','%.1f'));  set(gca,'YTickLabel',num2str(get(gca,'YTick')','%.1f'));  set(gca,'ZTickLabel',num2str(get(gca,'ZTick')','%.1f'));  hold on  axis([0 1 0 1 0 1])  end  end  end  end  xlabel('$e$','interpreter','latex');  ylabel('$f$','interpreter','latex');  zlabel('$g$','interpreter','latex','Rotation',360)；  %Array3  clc;clear;  Ce=5,Cf=25,Cg1=30,Cg2=0,Ch=20,Ff=0,Fg=10,Fh=0,Ig2=10,Ig1=10,Sh=6,Sg=12,Dg=24;  figure(1)  for i=0.1:0.2:1  for j=0.1:0.2:1  for k=0.1:0.2:1  for l=0.1:0.2:1  [t,y]=ode45(@(t,y) FP(t,y,Ce,Cf,Cg1,Cg2,Ch,Ff,Fg,Fh,Ig1,Ig2,Sh,Sg,Dg),[0 50],[i j k l]);  grid on  plot3(y(:,1),y(:,2),y(:,3),'linewidth',1);  set(gca,'XTick',[0:0.2:1],'YTick',[0:0.2:1],'ZTick',[0:0.2:1])  set(gca,'XTickLabel',num2str(get(gca,'XTick')','%.1f'));  set(gca,'YTickLabel',num2str(get(gca,'YTick')','%.1f'));  set(gca,'ZTickLabel',num2str(get(gca,'ZTick')','%.1f'));  hold on  axis([0 1 0 1 0 1])  end  end  end  end  xlabel('$e$','interpreter','latex');  ylabel('$f$','interpreter','latex');  zlabel('$g$','interpreter','latex','Rotation',360)；  %Array4  clc;clear;  Ce=15,Cf=25,Cg1=30,Cg2=0,Ch=20,Ff=0,Fg=10,Fh=0,Ig2=10,Ig1=10,Sh=6,Sg=12,Dg=24;  figure(1)  for i=0.1:0.2:1  for j=0.1:0.2:1  for k=0.1:0.2:1  for l=0.1:0.2:1  [t,y]=ode45(@(t,y) FP(t,y,Ce,Cf,Cg1,Cg2,Ch,Ff,Fg,Fh,Ig1,Ig2,Sh,Sg,Dg),[0 50],[i j k l]);  grid on  plot3(y(:,1),y(:,2),y(:,3),'linewidth',1);  set(gca,'XTick',[0:0.2:1],'YTick',[0:0.2:1],'ZTick',[0:0.2:1])  set(gca,'XTickLabel',num2str(get(gca,'XTick')','%.1f'));  set(gca,'YTickLabel',num2str(get(gca,'YTick')','%.1f'));  set(gca,'ZTickLabel',num2str(get(gca,'ZTick')','%.1f'));  hold on  axis([0 1 0 1 0 1])  end  end  end  end  xlabel('$e$','interpreter','latex');  ylabel('$f$','interpreter','latex');  zlabel('$g$','interpreter','latex','Rotation',360)； |
